# Draft Genome Sequences of Itaconate-Producing *Ustilaginaceae*

**DOI:** 10.1128/genomeA.01291-16

**Published:** 2016-12-15

**Authors:** Elena Geiser, Florian Ludwig, Thiemo Zambanini, Nick Wierckx, Lars M. Blank

**Affiliations:** Institute of Applied Microbiology—iAMB, Aachen Biology and Biotechnology—ABBt, RWTH Aachen University, Aachen, Germany

## Abstract

Some smut fungi of the family *Ustilaginaceae* produce itaconate from glucose. *De novo* genome sequencing of nine itaconate-producing *Ustilaginaceae* revealed genome sizes between 19 and 25 Mbp. Comparison to the itaconate cluster of *U. maydis* MB215 revealed all essential genes for itaconate production contributing to metabolic engineering for improving itaconate production.

## GENOME ANNOUNCEMENT

The members of the family of *Ustilaginaceae*, belonging to the phylum *Basidiomycota*, are known to naturally produce many different industrially interesting compounds, such as organic acids, lipids, and polyols (1–6). Previous bioprospecting utilizing glucose or glycerol identified several itaconate-producing *Ustilaginaceae* (1, 7). The investigated species and strains varied in their product spectra and the amounts of product. Among the species *Ustilago maydis*, individual strains differed highly in their ability to produce organic acids, although the product spectra did not differ. Some of the species investigated, for example *Ustilago vetiveriae* CBS 131474, produced itaconate only with glycerol as carbon source (1, 7). Also, the pH dependency of itaconate production varied a lot. While in *U. maydis* itaconate production is only possible in the pH-range of 5 to 7, *Ustilago cynodontis* strains can tolerate pH values as low as 3 (1). To gain a deeper understanding of the sequence-function relationship between itaconate genes (8) and itaconate production, the genomes of 12 *Ustilaginaceae* with different itaconate production levels were sequenced. The itaconate clusters of *Sporisorium iseilematis-ciliati* BRIP 60887 a, *Pseudozyma tsukubaensis* NBRC 1940, *Pseudozyma hubeiensis* NBRC 105055, *Ustilago vetiveriae* CBS 131474, and *Ustilago maydis* strains AB33P5ΔR, ATCC 22892, ATCC 22899, ATCC 22901, and ATCC 22904, were compared, as well as *Ustilago xerochloae* CBS 131476 and *Ustilago cynodontis* NBRC 9727 and CBS 131467 (data not shown).

Here, we present the draft genome sequences of these *Ustilaginaceae*. Genomic DNA (gDNA) was isolated by standard phenol-chloroform extraction (9). Eurofins Genomics (Ebersberg, Germany) did the library creation using an NEBNext Ultra DNA Library prep kit for Illumina (Art no. E7370) and sequencing by using an llumina HiSeq2500 machine with TruSeq SBS kit v3, both according to manufacturer’s instructions. Sequencing mode was 1 × 100 and the software used was HiSeq Control Software 2.0.12.0 RTA 1.17.21.3 bcl2fastq-1.8.4. Quality check of the sequence data was performed with FastQC (Version 0.11.2). The SPAdes-3.7.0-Linux pipeline was used for *de novo* genome assembly of single-read libraries and read error or mismatch correction, including BayesHammer, IOnHammer, SPAdes, MismatchCorrector, dipSPAdes, and truSPAdes. The k-mer size was determined to 55 using VelvetOptimiser Version 2.2.5. Parameters of the resulting sequences are summarized in Table 1.

**TABLE 1:** Genome sequence parameters

Strain	Reference or source	NCBI GenBank accession no.^*a*^	Sequence size (bp)	No. of large contigs (>300 bp in size)	G+C content (%)	Avg contig sequence size *N*_50_ (bp)	Max contig sequence size (bp)
*Ustilago maydis* AB33P5ΔR	11	LZQU00000000	19,929,430	1,978	50	111,545	354,120
*Sporisorium iseilematis-ciliati* BRIP 60887 a	Culture collection of the Queenslad Plant Pathology Herbarium (BRIP), Australia	MJEU00000000	23,207,148	15,412	54	128,139	422,913
*Pseudozyma tsukubaensis* NBRC 1940	12	MAIP00000000	23,769,677	12,840	52	161,943	632,715
*Pseudozyma hubeiensis* NBRC 105055	13	MAIO00000000	21,322,328	9,793	54	260,601	1,046,041
*Ustilago vetiveriae* CBS 131474	13	MAIM00000000	19,606,533	5,932	52	160,125	620,444
*Ustilago maydis* ATCC 22892	1	LYOO00000000	20,622,051	4,767	50	104,350	527,495
*Ustilago maydis* ATCC 22899	1	LYZD00000000	20,208,930	2,938	51	109,028	306,704
*Ustilago maydis* ATCC 22901	1	LZNJ00000000	20,063,391	2,505	51	102,739	354,117
*Ustilago maydis* ATCC 22904	1	LZQT00000000	20,132,962	2,695	49	120,656	551,297

a Versions described are the first versions.

By comparison to the itaconate gene cluster of *U. maydis* MB215 (8) (GenBank Accession Number KT852988), the complete itaconate cluster was identified in all sequenced organisms except *Pseudozyma tsukubaensis* NBRC 1940, which did not contain *rdo1* and *cyp3*, which encode a putative dioxygenase and a monooxygenase involved in OH-paraconate production, respectively (10). The synteny of the itaconate cluster is preserved in the investigated *Ustilaginaceae*. The sequences will foster research on the biology of *Ustilaginaceae* and increase the list of tools for metabolic engineering of itaconate production by *Ustilaginaceae*.

### Accession number(s).

The whole-genome sequences have been deposited in DDBJ/ENA/GenBank. Their accession numbers and version numbers described in this paper are listed in Table 1.
